# Sea Ice Microorganisms: Environmental Constraints and Extracellular Responses

**DOI:** 10.3390/biology2020603

**Published:** 2013-03-28

**Authors:** Marcela Ewert, Jody W. Deming

**Affiliations:** School of Oceanography, University of Washington, Campus Mailbox 357940, Seattle, WA 98195, USA; E-Mail: jdeming@uw.edu

**Keywords:** sea ice, bacteria, extracellular polymeric substances, halophiles

## Abstract

Inherent to sea ice, like other high latitude environments, is the strong seasonality driven by changes in insolation throughout the year. Sea-ice organisms are exposed to shifting, sometimes limiting, conditions of temperature and salinity. An array of adaptations to survive these and other challenges has been acquired by those organisms that inhabit the ice. One key adaptive response is the production of extracellular polymeric substances (EPS), which play multiple roles in the entrapment, retention and survival of microorganisms in sea ice. In this concept paper we consider two main areas of sea-ice microbiology: the physico-chemical properties that define sea ice as a microbial habitat, imparting particular advantages and limits; and extracellular responses elicited in microbial inhabitants as they exploit or survive these conditions. Emphasis is placed on protective strategies used in the face of fluctuating and extreme environmental conditions in sea ice. Gaps in knowledge and testable hypotheses are identified for future research.

## 1. Introduction

Sea ice is a dynamic, porous matrix that harbors within its interior network of brine pores and channels an active (e.g., [1,2]) and diverse [3,4,5,6] community. The sympagic (ice-associated) community has multiple trophic levels including photosynthetic bacteria and algae, chemoautotrophic bacteria and archaea, and heterotrophic bacteria, archaea, flagellates, fungi and small metazoans [5,7,8,9,10,11]. Members of this community, particularly the bacteria and algae, play important roles in cycling carbon [12,13] and nitrogen [14,15] in polar regions; selected bacteria also respond to pollutants such as crude oil [16,17] and mercury [18,19].

**Figure 1:**
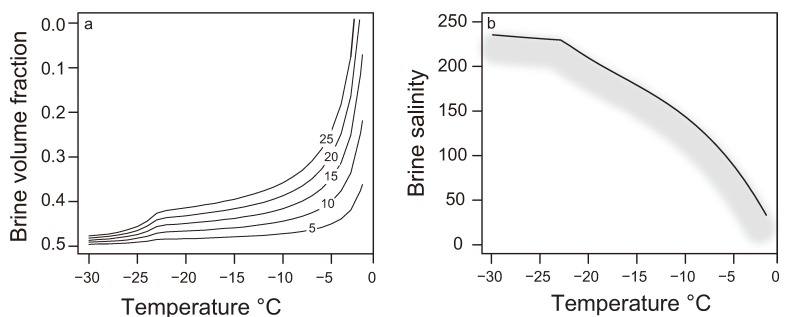
Dependence of brine volume fraction (**a**) and brine salinity (**b**) on sea-ice temperature, according to phase equations from Cox and Weeks [21]. Contour lines in (**a**) indicate the effect of different bulk salinities on brine volume fraction. Brine salinity (**b**) is independent of bulk ice salinity, conventionally determined only by temperature; we suggest, by the shadowing of the line, that the presence of extracellular polymeric substances (EPS) produced by sea-ice organisms may influence brine salinity in as yet unpredictable ways (see Section 3.3).

The seasonal (autumnal) decrease in temperature that leads to the formation of sea ice in polar waters progressively reduces the liquid phase of the ice—the brine volume fraction—and consequently increases the concentration of solutes and particles in the brine. Phase equations of sea ice [20,21,22] are frequently used to estimate brine salinity and brine volume fraction based on the temperature of the ice and its bulk salinity (salinity after melting). Temperature determines solute concentration such that when the ice reaches a temperature of −5 °C, just ∼3 °C below the freezing point of seawater, the estimated brine volume fraction has decreased below 0.3 (even as low as 0.05 for ice with low bulk salinity; Figure 1a) and the estimated brine salinity has increased to nearly 100 (Figure 1b). At extreme winter temperatures, salt precipitation within the brine phase adds complexity to these constraints (see deflection points at −22.9 °C, the eutectic for hydrohalite, in Figure 1b). Organisms, previously at seawater temperature and salinity, are thus exposed to much lower temperatures, higher salinities, and reduced habitable space soon after entrapment in sea ice.

Other properties of sea-ice brines also show temperature-dependent changes. For instance, the solubility of biologically relevant gases, including CO_2_ and O_2_, decreases as the salinity of brines increases, translating to a general degassing effect throughout the winter season [23]. Subsequently, biological O_2_ consumption can further reduce O_2_ concentration in some sections of the sea-ice column leading to the development of microbial communities and processes that are favored by low oxygen conditions [14,15,24].

Summer brings a new set of changes. Melting of snow and then surface ice, and the consequent formation of melt ponds, exposes sea-ice microorganisms to salinities close to that of freshwater, or else flushes them back to the ocean through brine drainage [13,25]. Although disadvantageous to halophiles, this surface melting generates an environment suitable for the growth of a distinctive community of freshwater microorganisms [26,27]. Increased solar radiation in the spring and summer also promotes growth of photosynthetic organisms, further influencing sea-ice brine composition and prompting the secretion of protective screening and quenching compounds [28]. The onset of significant algal photosynthesis imposes additional challenges to the resident microbial community in the form of oxidative stress (by the increase of O_2_) or decrease in pH (by consumption of CO_2_ and consequent shifts in the carbonate chemistry of brine).

Some microorganisms have constitutive adaptations (expressed constantly) that allow them to thrive or survive under specific conditions of high salinity or low temperature. In a variable environment such as sea ice, however, acclimation mechanisms that allow a microorganism to function across a range of conditions may have an advantage over constitutive mechanisms finely tuned to an extreme, but relatively constant, environment. Microbial adaptations to sea ice may involve intracellular processes, membrane proteins or the secretion of extracellular polymeric substances (EPS). The latter can modify the immediate surroundings of the organism and its neighbors. The protective role of EPS, which tends to be gelatinous in nature, has been recognized since the earliest stages of sea-ice microbiology, as this account from 1918 by McLean [29] relates:

“It is a curious fact, and yet a well-known experience, to find that bacteria may live dormant in ice for prolonged periods, and that infection may be carried through ice, but it is not so generally recognized that some bacteria prefer to grow on ice. Microorganisms, as a rule, are capable of resisting a low temperature when their ordinary activities cease, and they tend, either as single units or in clusters, to throw out a mucilaginous protein substance for their protection.”

**Figure 2:**
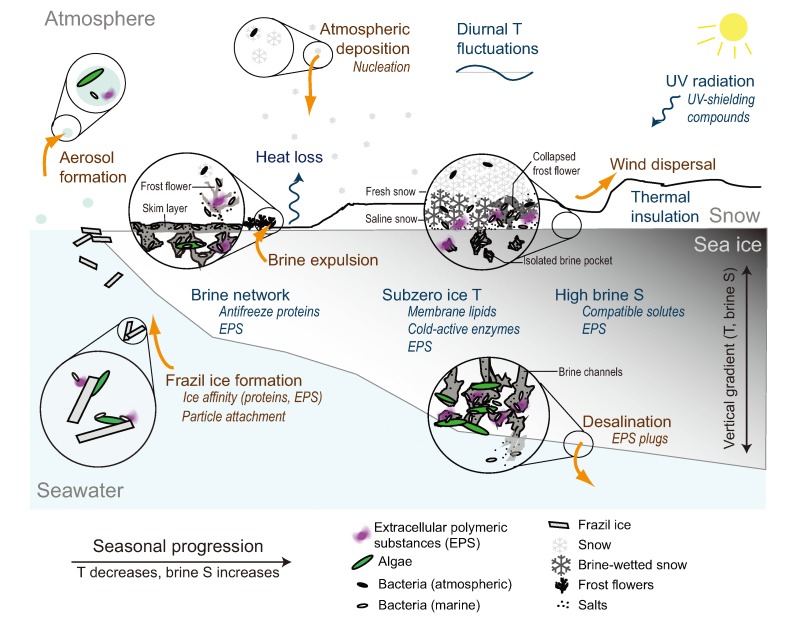
Schematic diagram of seasonal (fall through winter) processes influencing microorganisms in sea ice, including transport mechanisms (orange arrows) and some of the microbial adaptive responses (italics). During sea-ice formation, larger organisms ascend with rising frazil ice crystals; smaller bacteria and archaea likely attach to algae, particles or ice crystals. Once entrained in the ice, microorganisms inhabit a network of brine channels where they experience low temperature (T), high brine salinity (S) and reduced living space, but are protected from fluctuations in air temperature by the insulating properties of snow and ice. As sea ice consolidates, brines are expelled into the ocean (desalination) and onto the surface; a fraction of the microorganisms, EPS and other components of the brine are expelled, too. Surface-expelled brines and their contents form a skim layer that can be incorporated into frost flowers and snow, prone to wind dispersal. The skim layer and frost flowers, directly exposed to the atmosphere, experience more extreme fluctuations in temperature and brine salinity and, as the sun rises in late winter, greater UV exposure. From remaining areas of open water, including leads, wind can transport marine microorganisms in aerosols. Airborne microorganisms (including terrestrial bacteria) can nucleate snow and return to the ice/snow surface.

This review addresses some of the physico-chemical parameters that define the sea-ice environment on a seasonal basis and the protective responses they elicit in microorganisms, particularly bacteria, the category of microorganisms known to inhabit all depths, forms and seasons of sea ice (Figure 2). It diverges from prior reviews on sea-ice microorganisms and their environment (e.g., [7,30,31,32,33,34,35]) by giving particular attention to the protective role of EPS in the face of extreme fluctuations in key environmental parameters characteristic of the Arctic. In doing so, gaps in knowledge and new directions for research are identified.

## 2. Sea Ice as a Microbial Environment

### 2.1. Incorporation into Sea Ice

Sea ice formation starts in the fall. When water reaches its freezing point, ice crystals, known as frazil ice, form throughout the upper water column and rise to the surface accumulating in a slush. This surface slush continues to freeze, consolidating into an upper layer of ice. Ensuing sea-ice growth depends on the conditions of the ocean: calm conditions promote the formation of congelation ice; turbulent conditions lead to frazil ice growth [36]. The growth of sea ice produces a porous structure that, unlike glacial ice, retains abundant impurities that were present in the source water. Salts and other solutes, organic and inorganic particles, and microorganisms are rejected by the growing ice lattice into interconnected liquid inclusions within the ice. The liquid inclusions, brine channels and pores, form an interconnected network that accumulates high concentrations of solutes and constitutes the inhabited fraction of sea ice [37].

Different processes can contribute to the retention of some of the microorganisms, particles and organic substances entrained into the ice from the source water (Figure 2). Physical concentration can enrich algal cells, either through scavenging by frazil ice or by water circulation through the newly established ice layer [38]. Scavenging occurs when frazil ice crystals drift towards the surface, dragging in their path particles and algal cells from the water column that will later concentrate in the ice [39,40]. The physical concentration of algae has been observed in the field, with algal cells >10 µm preferentially enriched in young Arctic sea ice [8]. The ability of marine algae to nucleate ice crystals [41] could also contribute to the enrichment process of sea-ice algae by favoring the formation of ice crystals in their immediate environment that can lift them towards the consolidating ice layer.

In contrast, enrichment of smaller bacterial (and archaeal) cells in sea ice does not appear to occur directly by physical processes. Bacterial incorporation can be facilitated by the presence of algae, through bacterial association with algal cells or aggregated algal EPS, that are then concentrated by physical processes [38,42,43]. Attachment of bacteria to algae in young Arctic sea-ice samples has been observed but, because insufficient data are available to consider it a widespread phenomenon, the potential role of EPS is highlighted instead [43]. Indeed, particulate EPS (pEPS; > 0.4 µm) of likely algal origin are rapidly enriched in newly forming sea ice [43,44]. Bacterial EPS also have the potential to play a role in the entrainment and retention of bacteria in ice either directly or by promoting attachment to algal cells or detrital particles amenable to physical entrainment. Dissolved EPS (dEPS; < 0.4 µm) produced by *Colwellia psychrerythraea* strain 34H, a model marine psychrophilic bacterium [45], was found to be selectively retained in saline ice under experimental conditions [46]. Marine bacteria with the ability to produce an EPS coating with similar properties could be retained in the ice by this means alone, independently of association with algal cells or their byproducts, an hypothesis that remains to be tested.

As sea ice consolidates, brines in the upper layers of the ice are expelled upwards to the ice surface forming a surface skim layer (Figure 2). Sea-ice microorganisms (primarily bacteria), EPS and dissolved organic compounds are carried with the brine to this even colder habitat at the ice-atmosphere interface. A fraction of the bacteria and EPS may be selectively retained in the ice, however, following the arguments and potential mechanisms outlined for initial entrainment into the ice (EPS coatings and attachment to larger particles or ice crystals). Brines in this skim layer, and the bacteria and organic substances within it, can subsequently be incorporated into frost flowers that form on the new ice surface [47] or into the saline snow layer, which represents a vast bacterial habitat in its own right [48]. Frost flowers and saline snow will have brine inclusions with properties similar to those of the ice, but exposed to more extreme environmental parameters.

### 2.2. The Low Temperature Constraint

**Figure 3:**
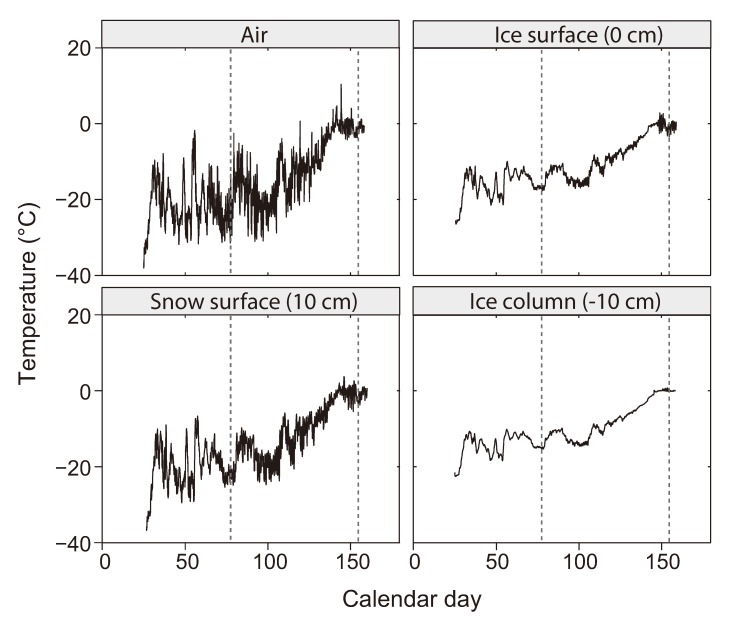
Temperature recorded at the Mass Balance Observatory Site (Barrow, AK, USA) during 2011 (days of year 25–158) at different depths above and below the ice surface. Dashed lines mark seasonal transitions. Spring equinox was on day 79, summer solstice on day 171.

A defining characteristic of the sea-ice environment is temperatures below, and sometimes well below, 0 °C. Meltponds, the accumulated meltwater from snow and surface ice during the summer season, are the only ice-associated environments with temperatures above 0 °C (between 0.4 and 1.5 °C, according to Lee and collaborators [13]). Sea-ice temperatures range typically between −2 °C and −30 °C with the coldest ones recorded in upper sea ice during Arctic winter [49] (winter lows for Antarctic sea ice are less extreme). Environments associated with the surface of new ice in winter, such as the brine skim layer and frost flowers, can be exposed to air temperatures below –30 °C. As a result of the insulating properties of ice and snow, environments experiencing the most severe fluctuations in temperature (and thus brine salinity) are those directly exposed to the winter atmosphere: the brine skim layer and frost flowers on the surface of new ice and, to a lesser degree, the saline snow layer when snow is blown to minimal thickness (as discussed by Ewert and collaborators [48]). To illustrate a typical range of temperatures and fluctuations seasonally experienced in Arctic sea ice and associated environments, Figure 3 presents detailed temperature measurements from the Mass Balance Observatory Site from the University of Alaska Fairbanks [50]. This observatory, located in landfast coastal sea ice in the coast of Barrow, Alaska (156.5° W, 71.4° N), measures air-snow-ice-water temperature profiles throughout the winter and spring seasons.

Low temperatures impose constraints at different levels in the single-cell microorganism and, consequently, elicit responses spanning different aspects of its physiology. Reaction and transport rates decrease with temperature, slowing most physiological processes. Protein folding is affected by a decrease in hydrophobic forces and changes in hydration [51]. Membranes become rigid. Nucleic acids become more stable, which hinders replication, transcription and translation processes. Microbial solutions to these constraints also span the gamut of possibilities. Cold-active enzymes remain functional at low temperatures by favoring amino acids that allow higher flexibility and structural modifications that provide ligands better access to the catalytic site [51]. Bakermans and collaborators [52] found that *Psychrobacter cryohalolentis* K5, a psychrotolerant bacterium isolated from permafrost, showed important changes in its proteome with up to 30% of the proteins having significantly different levels of expression when exposed to low temperatures. Among these proteins were cold-shock chaperones to facilitate translation, cold-adapted alleles that would allow a same function be performed at two different temperatures, and an increase in the expression of certain transporters. Bacteria also respond to low temperatures by changing the type of fatty acids and carotenoids present in their membranes and altering the membrane protein content [35,52,53]. In fact, some bacteria can perceive changes in temperature through a membrane-bound sensor that triggers the expression of cold-activated genes [53].

Given the prevailing low temperatures, organisms inhabiting sea ice and associated environments can be expected to use many of these strategies to cope with low-temperature constraints. When compared with the underlying water, the sea-ice bacterial community is enriched in culturable taxa considered psychrophilic, *i.e.*, uniquely adapted to low temperatures [54]. Also, psychrophilic organisms tend to be more abundant in the upper layers of the ice, which have been exposed to the coldest temperatures during the winter [54]. Extracellular enzymes from sea ice have also been recognized for their unusually low optimal temperatures [55], especially those assayed in winter ice [56].

### 2.3. The Brine Channel Network

The brine channel network containing the liquid fraction of the ice has a complex three-dimensional structure [57]. For relatively warm ice near the ice-water interface, brine channels range in diameter from a few to hundreds of micrometers; the network is dominated by the smallest channels (<40 µm) that account for about 50 % of the surface area [9]. Brine inclusions are characterized by their volume fraction and connectivity, temperature-dependent properties that determine the permeability of the ice. For ice with a given bulk salinity, the size and connectivity of the brine inclusions will decrease with temperature until the ice reaches a critical porosity where it is no longer permeable [58]. According to phase equations of sea ice, the threshold for fluid permeability in sea ice occurs when the porosity approaches 5%, which occurs at a temperature of about −5 °C for ice with a salt concentration of 5 ppt [59]. Once the ice reaches the permeability threshold, pockets of brine become isolated from the underlying seawater and from each other (though micrometer-scale connections remain possible [56]). Shrinking of brine inclusions leads to an increase in the concentration of salts and other solutes, and of organisms and other particles present in the brines. This temperature-dependent concentration of solutes exposes sea-ice organisms to seasonal changes in salinity. The uppermost section of the ice experiences the most drastic changes, where, as temperature decreases in the winter, the concentration of salts in the brine can reach a salinity of 220, or 6–7 times higher than that experienced by microorganisms in seawater before their entrapment in the ice. As salts in the brine concentrate above their saturation points, ice experiences the successive precipitation of ikaite (CaCO_3_·6H_2_O) at −2.2 °C, mirabilite (Na_2_SO_4_·10H_2_O) at −8.2 °C, and hydrohalite (NaCl ·2H2O) at −22.9 °C [20,60]. Salt precipitation changes the ionic composition of sea-ice brines with respect to the source seawater and generates additional solid surfaces (salt crystals) with which microorganisms can interact. Such interactions have not been explored, except conceptually (see Section 3.3).

Temperature-dependent reduction of brine volume also increases the percentage of brine channel area covered by organisms [9], as well as the concentration of bacteria, viruses and free DNA within the brine [61,62] simultaneously increasing their contact rates in exponential fashion [63]. High concentrations and contact rates with viruses and nucleic acids have been hypothesized to promote lateral gene transfer in sea ice [56,64]. Properties of the brine channel network, mainly its connectivity and volume fraction, also affect the type of predators and the predator-prey dynamics of the sea-ice community. In general, larger predators only access the lowermost sections of the ice [65], with pores of less than 200 µm considered a refuge for smaller organisms (microalgae, ciliates and bacteria [9]). Some metazoan predators such as rotifers and turbellaria, though, are flexible enough to squeeze into brine channels with diameters much smaller than their bodies, and also adjust their body size according to changes in ambient salinity [9]. In the smallest sea-ice brine inclusions, viruses take the role as main predators [11,61].

Observations by Krembs and collaborators [66] confirmed the role of EPS-producing microorganisms in directly influencing the properties of the brine channel network. The presence of EPS increased the abundance of pores in the ice by 15% (over EPS-free ice) and led to the formation of pores with convoluted irregular shapes [66]. The effects of EPS were also evident in the larger pores (> 250 µm), where perimeter-to-length ratios corresponded to a fractal geometry, as opposed to the Euclidean geometry characteristic of pores in artificially grown sea ice lacking EPS [66]. The presence of EPS thus affects the habitability of sea ice by increasing the volume of the habitable liquid phase and the interior ice-liquid surface area available for “colonization.” These effects result from interactions between EPS and the ice, whether by clogging the brine channel network, changing the viscosity of the brine or directly associating with the ice crystals [66].

Sea-ice microorganisms can also modify the brine channel network through their antifreeze proteins, another type of extracellular substance produced by both sea-ice diatoms [67] and bacteria [68]. Extracellular antifreeze proteins secreted by the sea-ice diatom *Fragilariopsis cylindrus* can alter the microscopic and macroscopic structure of saline ice, opening the possibility for this protein and similar ones to play an important role in shaping the sea-ice microbial environment if produced in sufficient quantities [69,70]. The presence of extracellular organic substances with the ability to change macroscopic and microscopic structure of sea ice suggests a possible need to re-evaluate the applicability of Cox and Weeks [21] equations to describe brine salinity and brine volume fraction in natural, EPS-rich sea ice (Figure 1a). The issue is of particular relevance since the phase equations of sea ice are a common tool for estimating the brine salinities experienced by sea-ice organisms *in situ*.

### 2.4. The Brine Salinity Constraint

The high salinity characteristic of sea-ice brine imposes at least two types of constraints on resident microorganisms. First, high concentrations of salts tend to affect the functioning of proteins, including precipitating them. Bacteria and archaea inhabiting high salinity environments tend to have, as a response, acidic proteins that, given their abundant negative charges, remain soluble and functional at higher salinities than basic proteins. Second, high environmental salinity exposes organisms to high osmotic pressure that drives water out of the cell, resulting in potential dehydration, loss of turgor pressure and reduction of cell volume. To counteract this water efflux, microorganisms of all types compensate for excessive concentrations of external solutes by accumulating compatible solutes in the interior of the cell. The general microbial ability to tolerate and even thrive in sea-ice brines comes with the added benefit of refuge against metazoan predators more susceptible to increases in salinity, such as those reported by Krembs and collaborators [9].

**Figure 4:**
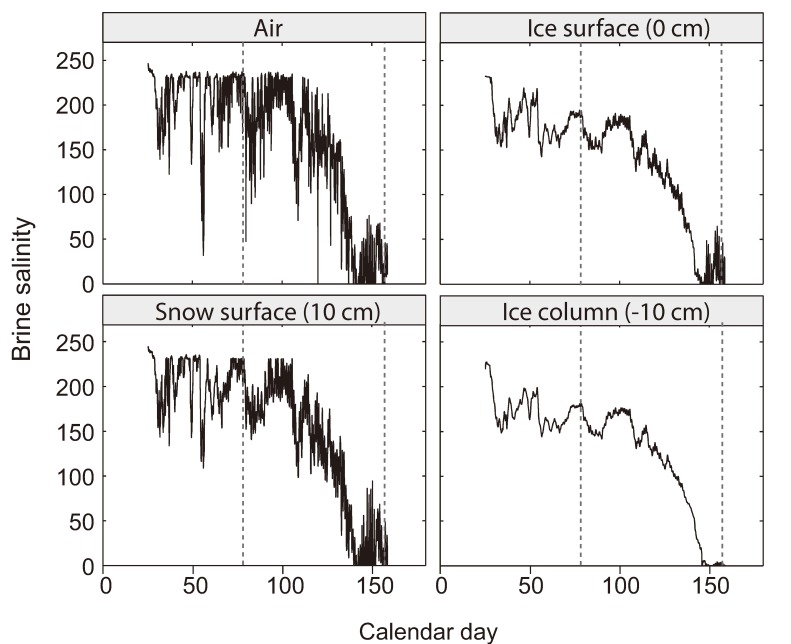
Brine salinity estimated from temperature data in Figure 3. Depths and dashed lines as in Figure 3. Brine salinity calculated using air temperature represents the extreme situation in which expelled sea-ice brines are directly exposed to the atmosphere and in thermal equilibrium with it.

The osmotic up-shift that occurs with ice formation happens quickly as the temperature of the ice drops (Figure 1b). Sea-ice brines, though, are distinguished from other high-salinity environments not only by subzero temperature but also by extreme fluctuations in salinity (Figure 4). A common bacterial response to osmotic up-shift starts with the transient accumulation of K^+^ and glutamate, accompanied by a release of putrescine to balance intracellular charges [71,72]. Avoiding growth limitations inherent to an intracellular accumulation of salts, microorganisms replace the accumulated K^+^ with compatible solutes, which are either imported or synthesized directly in the cell [71]. Compatible solutes are small, water-soluble organic molecules that increase the osmolarity of the cytoplasm without the disruptive effect of salt ions [73]. Dozens of compatible solutes have been described for Bacteria and Archaea, including free amino acids and their derivatives, sugars and their derivatives, and polyols and their derivatives. Among the most common compatible solutes are betaine, ectoine, trehalose, α-glucosylglycerol and glutamate [73]. A suite of genes allows for the transport of compatible solutes from the environment and/or their synthesis in the cell [74].

Not all organisms accumulate compatible solutes. Some extremely halophilic microorganisms, such as *Halobacterium salinarum*, which grows optimally at 26% NaCl, compensate high external concentration of solutes by incorporating salts in their cytoplasm. To keep cytoplasmic proteins functioning after the accumulation of salts, *H. salinarum* expresses an unusually high ratio of acidic to basic proteins (4.9 for the complete proteome [75]). Membrane proteins, adapted to function when directly exposed to the high-salinity environment, present a similar tendency to be acidic independently of the intracellular accumulation of organic solutes or K^+^ salts in the cytoplasm [76].

Partial proteomes available to examine for bacteria known from sea ice, when compared with bacteria and archaea from other saline and fresh-water environments (following [76]), also present the signature of a high acid-to-basic ratio in their membrane proteins (Table 1), consistent with the high salinities seasonally experienced in sea-ice brines. Their cytoplasmic ratios, however, do not compare with the extremely halophilic reference strain. Although the difference may be domain-specific (*Halobacterium*, contrary to its name, is an archaeal genus), we hypothesize that the salting-in strategy used by *H. salinarum*, with long term accumulation of K^+^ ions in the cytoplasm and the majority of cytoplasmic proteins being acidic, has not been adopted by microorganisms from the sea-ice environment despite exposure to high brine salinities. Greater physiological flexibility will be provided by use of the compatible solutes strategy in the face of strong seasonal fluctuations in salinity inherent to sea-ice brines. Note that *Psychromonas ingrahamii*, which has the lowest ratio of acidic to basic proteins in either its membrane or cytoplasmic proteome compared with other marine isolates considered (Table 1), was isolated from the sea ice/water interface, which tends to have lower salinities and species not necessarily adapted to life in the brine channel network [77].

Extracellular polymeric substances are also used by sea-ice microorganisms as a response to elevated salinities. The sea-ice diatom *Fragilariopsis cylindrus* has been shown to increase the production of all types of EPS (soluble, insoluble and frustule-associated) when frozen under high-salinity conditions [77]. Likewise, high concentrations of EPS from a sea-ice isolate of the bacterial genus *Pseudoalteromonas* were shown to extend the range of salinities at which this strain could grow, while also providing protection against freeze-thaw cycles [78].

Osmotic down-shift will be experienced in the late spring and the summer as the ice warms and melts. The extent of the osmotic down-shift depends on summer brine drainage. If melting prompts the brine to be flushed back into the ocean, microorganisms may not likely experience salinities much lower than seawater. If melting results in the formation of surface meltponds, then one of two conditions will follow: meltponds connected to seawater will have salinities close to 29, similar to those in nearby surface water; unconnected meltponds will have salinities below 5, reaching values as low as 0.1 [13]. In the latter case, microorganisms will be exposed to a drastic down-shift in salinity, which could result in the lysis of a significant fraction of the population. For bacteria, even if not directly lysed, a down-shift may prompt lysogenic viruses (already carried by the cell) to enter the lytic stage in those cells with an active metabolism [79,80] and lead to bacterial loss by that mechanism. The possibility that an EPS coating may protect against a drastic down-shift in salinity or viral lysis has not been tested (see Section 3.2).

**Table 1:** Ratio of acidic to basic proteins in partial proteomes of selected microorganisms.

Organism	Membrane	Cytoplasmic	Environment
Extremely halophilic			
*Halobacterium salinarum*	3.88	16.8	Highly saline lakes
Halophilic			
*Psychrobacter cryohalolentis*	2.22	3.27	Cryopeg
*Roseobacter denitrificans*	2.22	3.00	Marine
*Psychrobacter arcticus*	2.14	3.34	Permafrost
*Sphingopyxis alaskensis*	1.90	2.23	Marine
*Shewanella frigidimarina*	1.52	2.90	Marine, sea ice
*Colwellia psychrerythraea*	1.47	3.17	Marine sediments, sea ice
*Shewanella oneidensis*	1.47	3.26	Anaerobic sediments
*Marinobacter aquaeolei*	1.42	3.13	Marine
*Oceanobacillus iheyensis*	1.15	3.78	Marine sediments
*Psychromonas ingrahamii*	1.10	2.00	Sea ice / water interface
Non-halophilic			
*Flavobacterium psychrophilum*	0.75	1.44	Freshwater fish
*Lactococcus lactis* subsp. lactis	0.74	4.03	Gut flora
*Sphingomonas wittichii*	0.69	2.28	River

Ratio of proteins with isoelectric point (pI) < 7 to proteins with pI > 7; pI calculated with the *Compute pI/Mw* tool from the ExPasy Bioinformatics Resource Portal [81]. All reviewed protein entries for each organism retrieved from the UniProtKB data base on November 2012 [82], annotated for location as either “membrane” or “cytoplasmic.”

### 2.5. Insolation

Solar radiation drives numerous reactions, biotic and abiotic, including the alteration and destruction of biologically relevant molecules. Processes driven by solar radiation, and the responses they trigger in microorganisms, are of particular relevance in polar regions where strong seasonal changes in insolation occur. At the organism level, UV radiation (UVR) can damage DNA and other nucleic acids by the formation of thymine dimers. UVR is known to decrease viability in bacteria from aquatic ecosystems [83] and damage the photosynthetic potential of benthic algae [84].

Given the potential detrimental effects of high irradiation, microorganisms have developed multiple protective responses, including the production of shading pigments, antioxidant compounds, and the performance of rapid DNA repair. For instance, in sediment-associated diatoms, exposure to UV-B prompts motility (away from radiation) and the production of carotenoid pigments able to function as quenching agents [84]. The synthesis of mycosporine-like amino acids (MAA), a type of UVR-screening compound, is widespread in marine microscopic algae, especially those associated with surface blooms [85]. Similar responses to UVR are found in the sea-ice microbial community. Uusikivi and collaborators measured relatively high concentrations of MAA in Baltic sea ice, particularly in the surface layers [86]. Likewise, Mundy and collaborators reported the production of carotenoid pigments and mycosporine-like amino acids by algal communities associated with sea ice during the melting season of Arctic coastal first year ice, under high levels of UVR [28]. Motility by sea-ice algae in response to changing irradiance has been suggested [87] but, to the best of our knowledge, has not been confirmed as a mechanism of photoadaptation in sea ice. The general effects of UVR on EPS are less clear, with some (non-sea-ice) studies finding an increase [83] and others a decrease [84] in EPS content of the UV-exposed community.

Solar radiation further influences the sea-ice ecosystem by driving reactions that modify the dissolved organic carbon (DOC) pool. The potential of organic compounds to participate in photochemical reactions can be inferred from their absorption of visible radiation and UVR. DOC from spring sea ice, known to absorb UVR, has been shown to undergo varied photochemical reactions including changes in bioavailability and photooxidation to CO2 [23,88]. EPS are also affected by solar radiation. Ortega-Retuerta and collaborators demonstrated that transparent exopolymer particles (an alternative descriptor for pEPS) from natural North Sea water and from cultures of the marine diatom, Chaetoceros affinis, can be photolysed by UV-B (290–315 nm), and to some extent by UV-A (315–400 nm) and photosynthetically active radiation (400–700 nm) [89].

**Figure 5:**
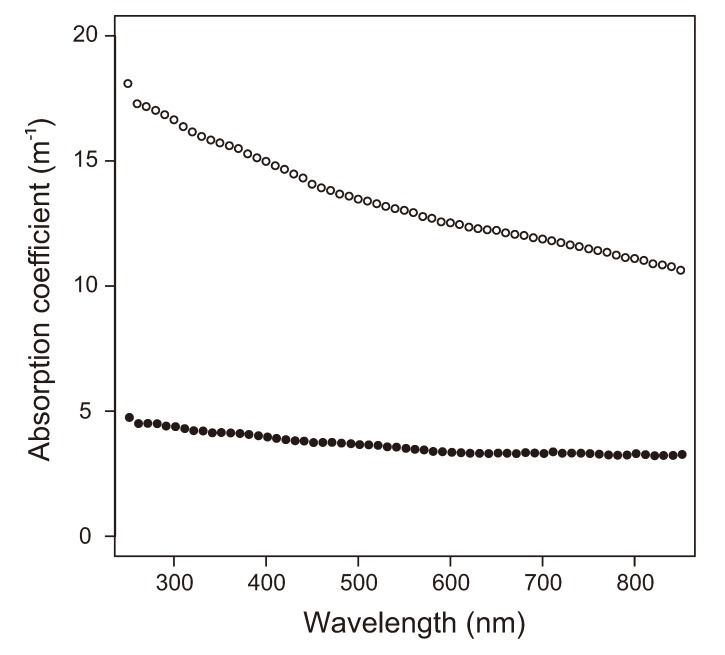
Absorption spectra for pEPS solution concentrated from surface samples of winter first year ice (open circles, 13 mg glu-eq mL^−1^) and saline snow (filled circles, 9.3 mg glu-eq mL^−1^). Samples were collected offshore Barrow, Alaska, in February 2010, filtered onto 0.4 µm polycarbonate filters as described by Ewert and collaborators [48], kept frozen in the dark at −20 °C for 20 months, and resuspended in 1.5 mL of distilled water for analysis.

Possible effects of solar radiation on EPS specific to sea-ice environments have been considered [47] but not tested to our knowledge. As part of a larger study [48], we examined the susceptibility of Arctic sea-ice EPS to photochemical reactions by measuring absorption spectra for pEPS samples from upper sea ice and saline snow. Absorption, converted to Napierian absorption coefficients (m−1), was higher in the UV-B range (Figure 5), suggesting that EPS associated with the winter sea-ice bacterial community may be susceptible to photochemical changes during the spring and summer when the radiation level increases. Similar profiles to the one in Figure 5 have been observed for particulate organic matter from late-winter surface Baltic sea ice [86], except for a peak in the 320–345 range associated with MAA that was absent from our samples. Samples from Baltic sea ice [86] were collected after the snow melt and contained a community of microscopic algae likely responsible for the production of MAA; in contrast, our samples were collected before snow melt and dominated by a bacterial community, explaining the absence of a MAA signature. In fact, Cockell and collaborators found that a snow cover of 5–15 cm thickness could reduce the transmittance of UV by an order of magnitude and reduce the impact of radiation in bacterial spores [90]. The snow cover over sea ice may thus act as a seasonal shading agent, protecting surface sea-ice microorganisms against UV radiation. This protective cover, though, is highly heterogeneous in thickness and melts early in the season [91].

## 3. Extracellular Responses to Sea-Ice Environmental Constraints

### 3.1. Extracellular Polymeric Substances

Extracellular polymeric substances (EPS), composed primarily of polysaccharides, are commonly produced by a wide range of microorganisms from both terrestrial and marine environments. EPS differ amongst organisms and producing conditions in sugar chain length and branching, sugar composition, type of sugar linkages, and the presence of additional chemical groups such as sulfates, proteins, lipids and even nucleic acids [92,93]. Different bacterial strains can produce EPS of different chemical composition and structure [93,94], but a single strain can also produce more than one kind of EPS [95]. Likewise, the type and amount of EPS produced by a bacterial strain can be modified by exposure to certain environmental conditions such as salinity [96], temperature [97] or presence of heavy metals [98]. Hence, the term EPS does not refer to a single chemically defined molecule but a complex mixture of diverse polysaccharides and ancillary compounds. Because EPS measurements typically quantify only the polysaccharide fraction of these components, the term EPS has also been used to refer specifically to extracellular polysaccharide substances. We use EPS throughout this review in its broadest meaning, unless otherwise specified.

EPS can be either tightly bound to the cell surface, loosely attached, or cell free [99]. Cell-free hydrophobic EPS from mesophilic bacteria have been shown to self-assemble into polymer microgels and to accelerate the self-assembly of microgels in seawater, with implications for concentrating organic-rich substrates for bacterial degradation [100]. These properties, however, were inhibited at low temperature for the particular polysaccharides studied [100]. The extent to which such self-assembly may occur in the sea-ice environment, where solute concentrations are high and EPS may be derived from psychrophilic microorganisms, has not been fully explored. An initial analysis in winter ice indicated minimal self-assembly [101], yet EPS aggregates with spherical diameters between 2 and 50 µm have been observed in sea ice [44].

### 3.2. EPS in Sea Ice

Most of the EPS in sea ice can be attributed to production by ice algae, either before or after entrainment into the ice. Even in regions of the ice dominated by bacteria, the trail of algal EPS is expected to overwhelm the amount produced by sea-ice bacteria [11,66]. Diatoms may produce distinctive EPS depending on their particular sea-ice habitat. Two diatoms isolated from the sea-ice brine network, *Fragilariopsis curta* and *F. cylindrus*, produced complex polysaccharides of higher molecular mass, with low relative abundance of glucose but high relative content of galactose, xylose and fucose. In contrast, a species of *Synedropsis* from the ice-water interface produced EPS dominated by low-molecular weight polysaccharides with low complexity and high relative content of glucose [77].

EPS abundance in sea ice and associated environments has been quantified in numerous studies beginning with those by Krembs and collaborators in 2002 [101] and by Meiners and collaborators in 2003 [44]. During fall, the number of EPS particles in sea ice can be an order of magnitude higher than in underlying water and often correlates with the presence of sea-ice algae [44]. The dissolved EPS fraction is consistently more abundant in sea ice [66] and sea-ice associated environments such as frost flowers [102] and saline snow [48].

The EPS pool in sea ice is established during ice formation [43,46,102] but can be modified subsequently by the entrained microorganisms. For instance, sea-ice microorganisms can add EPS to the existing pool by producing it *in situ* as a stress response, a process inferred from the increase in EPS concentration in winter sea ice [49,101]. On the other hand, bacteria may selectively degrade and consume certain fractions of the EPS pool, changing its overall chemical composition and size fractionation, as suggested by the detailed analyses of Underwood and collaborators [103].

The widespread, yet heterogeneous (e.g., [103]), presence of EPS in sea ice and associated environments may reflect the varied functions these polymers perform at different ecosystem levels [99,104]. At the microorganism level EPS have been associated with cell adhesion and aggregation [105], motility [106], affinity for metals [107], and with providing a sticky framework to keep extracellular enzymes in the immediate vicinity of the cell [99]. EPS can also provide protection against toxic heavy metals [108] and desiccation [109]. All of these functions have relevance in sea-ice environments. In particular, recent experimental data have shown that EPS can play a role in protecting sea-ice bacteria [78] and diatoms [77] against the challenges of high-salinity brines. These results are in agreement with data from other environments where high-salinity stress triggered changes in the type and amount of EPS produced by microorganisms from anaerobic sludge [96] and by freshwater cyanobacteria [110]. Likewise, EPS could have a role in protection against low salinity shocks. The marine psychrophilic bacterium *Colwellia psychrerythraea* strain 34H, whose immediate relatives are found in sea ice, increased the amount of EPS produced per cell when exposed to low salinities not permissive of growth [111]. The survival benefit was implied but not directly tested.

### 3.3. Influence of EPS on Physical-Chemical Properties of Sea Ice

Further insight into the protective role of EPS comes from experiments by Krembs and collabora­tors [66], who observed that artificial ice formations containing algal EPS had higher bulk salinities than EPS-free counterparts. This result has been related to the potential of EPS to form “plugs” in the brine channels, increasing the amount of salts that are retained (Figure 2). Following the phase equations of sea ice, higher bulk salinities result in higher brine volume fractions under similar temperature regimes (Figure 1b), effectively increasing the available habitable space for microorganisms.

The salinity of the brine pockets, however, is conventionally described as a function of temperature only and does not depend on the bulk salinity of ice. Following earlier work [66,112], we suggested in Figure 1b that the presence of EPS may have an effect on the validity of traditional phase equations when applied to natural sea ice. Some possible mechanisms may involve extracellular polymers (whether EPS or proteins [46]) with ice activity interacting with the ice surface of the brine pores and channels. If an important fraction of the surface area is covered, the growth of ice crystals might be restricted, resulting in local areas with lower salinity than predicted. EPS may also partition the brine within an ice pore creating microscale salinity gradients that affect ice crystal growth in currently unpredictable ways [112]. Another option could be antifreeze proteins, whereby more water in the liquid state would mean lower salinities. An EPS plug physically decreasing the minimal size of the brine pocket would have a similar effect.

Divalent cations present in sea-ice brines can also interact with charged groups in the backbone of EPS. In the marine environment, this interaction has been suggested to play a role in the binding of key nutrients for the cell such as iron [113]. Likewise, the interaction of EPS with Ca^2+^ determines self-assembly of marine gels, which can in turn increase the availability of nutrients for the microbial population [114]. In the case of sea ice, the relationship between EPS and Ca^2+^ may figure in the fate of carbonates in sea ice [115]. Relationships between bulk measures of dissolved organic matter and CaCO_3_ precipitation were not evident in Antarctic sea ice [116], but experiments specifically using EPS under ice-brine conditions have not been reported. If the dissolved organic matter measured in seawater by Chave and Suess [117] included EPS, then evidence exists for a role in delaying the onset of CaCO_3_ precipitation. Data on the interactions between EPS and calcium in other environments [118] may inform first tests of this hypothesis for sea ice.

Bergmann and collaborators used conductometric titrations to estimate the amount of binding sites for divalent cations present in ionic and nonionic bacterial extracellular polysaccharides [119]. Conductometric titrations [120] measure changes in conductivity resulting from the addition of a saline solution to a solution of interest, and provide information on the charge density of polyelectrolites such as ionic polysaccharides. A non-ionic polysaccharide such as dextran has a titration curve where no interaction with Ca^2+^ ions is evident. Xanthan, being an ionic polysaccharide, has a titration curve with a clear offset due to its conductive properties and the presence of associated counter-ions. Its curve also shows two segments with distinctive slopes, indicating that Ca^2+^ ions interacted with the polysaccharide until all binding sites were occupied [119].

**Figure 6:**
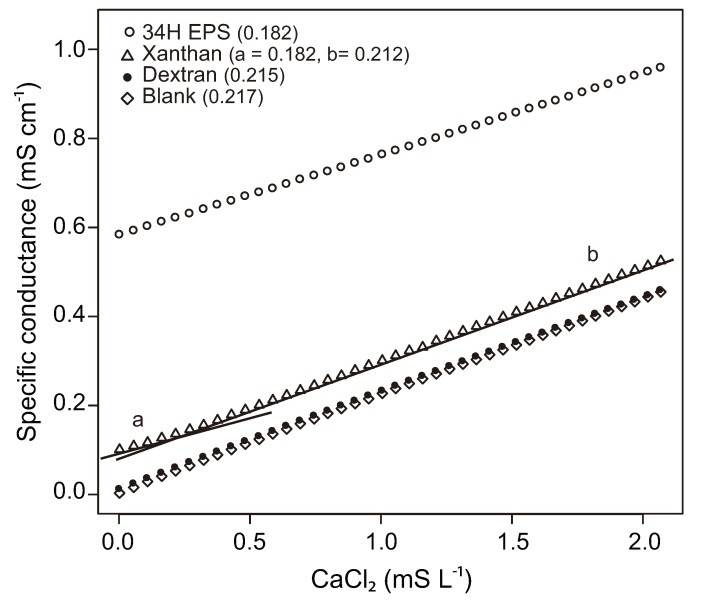
Conductometric titration of polysaccharide solutions with CaCl_2_ (0.05 M). Each data point shows the effect of increasing concentration of CaCl_2_ on preexisting solutions of polysaccharide (0.5 g L^−1^). Value in parentheses is the slope of the titration curve. Slopes were calculated using linear regressions, all of which have R > 0.99 and *p* value < 0.001. Experiments were performed at room temperature, with less than 1 degree difference among treatments (blank, 22.0 °C ± 0.1; dextran, 22.1 °C ± 0.1; xanthan 22.0 °C ± 0.1; 34H EPS, 22.9 °C ± 0.1). Cell-free EPS from strain 34H was extracted by centrifugation and precipitation with ethanol as in [111], followed by freeze-drying.

Following this approach, conductometric titrations were performed on solutions (in de-ionized water) of dextran, xanthan, and EPS obtained from a culture of *Colwellia psychrerythraea* strain 34H. A blank with no polysaccharide added was also included (see Figure 6 for details). The resulting titration curves and the slopes of their respective linear regressions (Figure 6) agree with results from Bergmann and collaborators [119]. The titration curve of dextran and the blank closely resemble each other, whereas the titration curve of xanthan has an offset and two segments with distinctive slopes. The slope of the 34H EPS curve is the same as the slope of the first segment for xanthan, the segment where interaction with Ca^2+^ is expected; there is no change in the slope, however, and the offset is 6 times higher. EPS from strain 34H thus likely contains charged polysaccharides with abundant backbone charges and associated counter-ions (high curve offset) and multiple binding sites for Ca^2+^ (no change in slope over the tested range of CaCl_2_ concentrations). The presence of charged EPS from this Arctic marine psychrophile has implications for the dynamics of carbonates in the sea-ice environment given that charged polysaccharides, unlike non-ionic polysaccharides, have known effects on the precipitation of carbonates [121].

Interactions between cations and polysaccharides also confer both algal and bacterial EPS with the potential to adsorb heavy metal contaminants such as Cd^2+^ [98], Pb^2+^ [122] and Hg^2+^ [123], which can then be incorporated into the food chain [124,125]. Disconcerting concentrations of these heavy metals have been found in the Arctic marine food web [126]; EPS from sea-ice organisms may be playing a role in the fate of these contaminants. Of special interest is the dependence of heavy metal adsorption to EPS on properties such as salinity, pH and Ca^2+^ concentrations [122,123,124], properties that undergo seasonal changes in sea-ice brines.

EPS can also interact with other extracellular macromolecules, most relevantly with proteins. Non-covalent interactions between EPS and proteins allow the formation of complexes, coacervates and aggregates, increasing the range of pH in which a protein is soluble [127]. In the case of cold-adapted marine organisms, EPS have been shown to increase the stability and half-life of a cold-active extracellular aminopeptidase from *C. psychrerythraea* 34H [128]. The interaction of EPS with antifreeze proteins is of particular interest for it could allow the accumulation of such proteins in the immediate vicinity of the cell, concentrating their antifreeze effects [70] to the benefit of sea-ice inhabitants as temperatures drop seasonally. Interactions between EPS and proteins under *in situ* conditions relevant to microbial life in sea ice remain largely unexplored.

## 4. Prospectus for Future Research

In focusing on the extracellular responses of sea-ice microorganisms to the sometimes severe and fluctuating environmental conditions of their habitat, our goal has been to highlight a number of features, particularly regarding EPS, that have not been fully explored or that raise new research directions. The extracellular products of microorganisms entrapped in sea ice are known to influence the microstructure of the ice, and thus its habitability, but how they may influence the effectiveness of traditional equations for calculating key parameters of sea ice or function at the micrometer scale within an ice pore is not clear. Unanswered questions involve the potential role of EPS in mitigating the salt concentration directly experienced by the cell, contributing to the adaptive strategy of compatible solutes, and blocking viral attack. EPS interactions with other exudates, including ice-active proteins and hydrolytic enzymes that serve a substrate-acquisition function for the cell, are poorly known. If the unexplored interactions of EPS with inorganic ions, particularly Ca^2+^, were to be as significant in sea ice as they are in other environments, then the implications for carbon transport through the sea-ice cover could be quantitatively important. As much as has been learned over the past decades about sea-ice microorganisms and their self-protective responses to the constraints of their habitat, more awaits discovery.
